# A52 OVERNUTRITION IN TREATED CELIAC DISEASE (CED) PATIENTS: THE NEED FOR PERSONALIZED NUTRITIONAL ASSESSMENT TO MANAGE THE METABOLIC SEQUELAE OF A GLUTEN-FREE DIET (GFD)

**DOI:** 10.1093/jcag/gwae059.052

**Published:** 2025-02-10

**Authors:** A K Verma, M Khaouli, F Abdi, J Blom, F J Echagüe, F Alessa, A Aman, D Armstrong, M pinto-sanchez

**Affiliations:** Medicine, McMaster University, Hamilton, ON, Canada; Medicine, McMaster University, Hamilton, ON, Canada; Medicine, McMaster University, Hamilton, ON, Canada; Medicine, McMaster University, Hamilton, ON, Canada; Medicine, McMaster University, Hamilton, ON, Canada; Medicine, McMaster University, Hamilton, ON, Canada; Medicine, McMaster University, Hamilton, ON, Canada; Medicine, McMaster University, Hamilton, ON, Canada; Medicine, McMaster University, Hamilton, ON, Canada

## Abstract

**Background:**

Overnutrition, leading to overweight and obesity, is increasingly prevalent in celiac disease (CeD). A nutritionally imbalanced diet may contribute to overnutrition and the development of Metabolic Syndrome (MS) and Metabolic Dysfunction Associated Steatotic Liver Disease (MASLD).

**Aims:**

To investigate the factors contributing to the metabolic shift in treated CeD.

**Methods:**

We enrolled patients with biopsy-proven CeD on a GFD and non-CeD (Inflammatory Bowel Disease and Irritable Bowel Syndrome) attending a tertiary care center adult Nutrition Assessment Clinic. We collected data on nutritional assessment (SGA), energy requirements determined by resting energy expenditure (REE: indirect calorimetry, QNRG, COSMED, US) and activity factor (IPAQ), body composition (3D scanner; Styku CA, US) and obesity risk (EOSS: Edmonton Obesity Staging System) to assess risk based on metabolic comorbidities such as diabetes, fatty liver, and cardiovascular disease. SPSS (version 22, US) was used for statistical analysis. Data are reported as median (IQR); Mann-U-Whitney was used for comparison between groups.

**Results:**

From November 2021 to October 2024, 124 CeD [Female: 76%; Age: 45 (27) yr; time since diagnosis: 5 (6) yr] and 96 non-CeD [F:74%; 48 (27) yr] patients were enrolled. Nutritional assessment identified undernutrition (BMI<19) in 8% vs 20%, overweight (BMI 26-30) in 24% vs 14%, and obesity (BMI>30) in 49% vs 34% of CeD vs non-CeD (p<0.001). REE adjusted for weight was significantly lower in obese CeD compared with non-obese CeD [17 (3) vs 22 (4) kcal/kg/day; p<0.001]. Fat mass was significantly greater [19 (7) vs 40 (10) kg; p<0.001], and fat-free mass was significantly reduced [FFM:43 (11) vs 54 (10) kg; p=0.005] in CeD with overweight/obesity compared to normal BMI. FFM was positively correlated with REE (r=0.71; p<0.001). Metabolic comorbidities occurred in 26% of obese-CeD patients. MASLD was more frequent in CeD with overweight and obesity compared to normal BMI (81% vs 43%; p=0.04).

**Conclusions:**

The high rate of overnutrition and reduced muscle mass in treated CeD patients is concerning, as this is associated with metabolic comorbidities. Personalized nutrition assessment with accurate measurements is crucial for nutritional guidance and will likely improve health in CeD.

**Funding Agencies:**

